# Precision-based modeling approaches for necrotizing enterocolitis

**DOI:** 10.1242/dmm.044388

**Published:** 2020-06-24

**Authors:** Mark L. Kovler, Chhinder P. Sodhi, David J. Hackam

**Affiliations:** 1 Division of Pediatric Surgery, Department of Surgery, Johns Hopkins University School of Medicine, Baltimore, MD 21287, USA; 2 McKusick-Nathans Department of Genetic Medicine, Johns Hopkins University School of Medicine, Baltimore, MD 21287, USA

**Keywords:** Necrotizing enterocolitis, Premature intestine, Toll-like receptor 4, Enteroid

## Abstract

Necrotizing enterocolitis (NEC) is the leading cause of death from gastrointestinal disease in premature infants and remains stubbornly difficult to treat in many cases. Much of our understanding of NEC pathogenesis has been gained through the study of highly translational animal models. However, most models of NEC are limited by their overall complexity and by the fact that they do not incorporate human tissue. To address these limitations, investigators have recently developed precision-based *ex vivo* models of NEC, also termed ‘NEC-in-a-dish’ models, which provide the opportunity to increase our understanding of this disease and for drug discovery. These approaches involve exposing intestinal cells from either humans or animals with or without NEC to a combination of environmental and microbial factors associated with NEC pathogenesis. This Review highlights the current progress in the field of NEC model development, introduces NEC-in-a-dish models as a means to understand NEC pathogenesis and examines the fundamental questions that remain unanswered in NEC research. By answering these questions, and through a renewed focus on precision model development, the research community may finally achieve enduring success in improving the outcome of patients with this devastating disease.

## Abstract (precis)

**Summary:** Much of our understanding of necrotizing enterocolitis (NEC) pathogenesis has been achieved through animal models. Here, we discuss the development of advanced precision-based models to improve outcomes for patients with NEC.

## Introduction

**Fig. 1.**
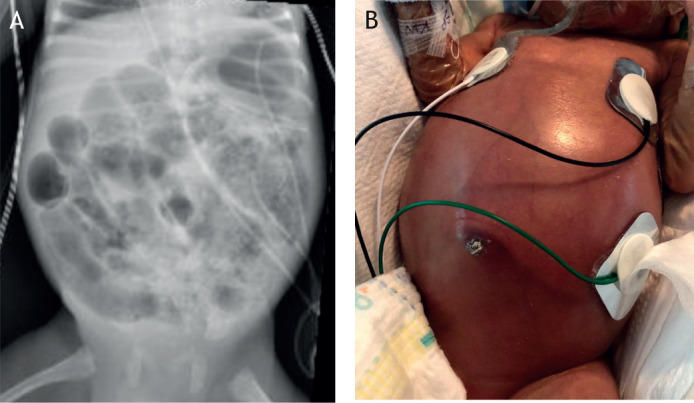
**Clinical features of NEC in a child.** (A) Abdominal x-ray of a premature baby showing extensive pneumatosis intestinalis. (B) Clinical appearance of a patient with NEC. Image credit: M.L.K.

Necrotizing enterocolitis (NEC) is the leading cause of death from gastrointestinal disease in premature infants, yet it remains a mystery to many physicians and a complete unknown to an even greater number of biomedical scientists (Stoll et al., 2015). The typical NEC patient is a premature infant in a neonatal intensive care unit who had been tolerating infant formula feeds, and then suddenly develops abdominal distention, bloody stools and rapid progression to overwhelming sepsis (Hackam and Caplan, 2018). At surgical exploration of the abdomen (laparotomy), the surgeon typically encounters patchy areas of intestinal necrosis involving the jejunum and ileum, accompanied by turbid intra-abdominal fluid and the presence of air within the wall of the bowel. This abnormal intestinal finding, termed pneumatosis intestinalis, may have been seen on plain abdominal x-ray imaging before rapid clinical deterioration occurs (Fig. 1), and reflects the accumulation of the products of gas-forming microbes in the gut wall (Smith et al., 2011). Clinical improvement frequently requires the resection of the dead or dying intestine and is successful in 60-70% of cases (Han et al., 2020). Unfortunately, the remainder of patients will progress to multi-system organ failure and death within hours or days of initial presentation (Moss et al., 2006; Alganabi et al., 2019). Although the above scenario describes the patient who quickly progresses from mild to severe disease, it is important to point out that there is a wide range of NEC presentations, and in fact many patients will have a less-severe course of disease. Some infants will have very mild disease characterized by abdominal distention and bloody stools. This milder course of NEC may readily resolve with antibiotics, cessation of feeds and fluid resuscitation alone (Hackam and Caplan, 2018). An even larger number of patients will have ‘possible NEC’, which can be subtle in presentation, and which may overlap with other infectious diseases in the neonatal period (Gephart et al., 2018). Given how quickly severe NEC develops, we have termed this clinical variant of NEC ‘staccato NEC’, which often reflects the presence of complete intestinal necrosis, or ‘NEC totalis’, and is associated with nearly 100% mortality (Sho et al., 2014).

While over 90% of patients with NEC have either prematurity or formula feeds as underlying risk factors, a substantial number will either be full term or will have been exclusively breast fed (Rose and Patel, 2018). Specifically, Gadepalli and colleagues have described the pathogenesis of NEC in full-term infants, and have identified the presence of cardiac disease as a significant underlying risk factor in this population (Overman et al., 2019). Moreover, Neu and colleagues have described the development of NEC in breastfed babies (Cacho et al., 2017), and have summarized that the risk of NEC is 2-5% in infants who are fed an exclusive human milk diet (Abrams et al., 2014), which confirms previous findings by Lucas and colleagues, who show a 6% incidence of NEC in breastfed infants (Sullivan et al., 2010). The reasons for NEC development in breastfed infants could be partly related to the variability in protective factors in breast milk. To this end, Bode and colleagues have shown that a relative paucity of the human milk oligosaccharide disialyllacto-N-tetraose was associated with NEC development in breastfed infants (Autran et al., 2018), providing a possible explanation for the observation that breastfed babies can sometimes develop NEC.

Despairingly, children who do survive the onset of NEC may develop persistent lifelong problems, including short bowel syndrome (Murthy et al., 2014), nutritional deficiencies that lead to small stature (Soraisham et al., 2006) and cognitive impairment accompanied by marked evidence of white-matter injury (Hickey et al., 2018; Robinson et al., 2018a; Shah et al., 2008). Despite its outsized impact on child health, many readers will have scarcely heard of NEC and even fewer will have devoted their research careers to studying it, perhaps explaining its stubbornly high mortality (Stoll et al., 2015).

The past several years, however, have yielded tremendous reason for optimism, in part due to an expansion of the tools available for studying NEC. First and foremost, these tools include clinically relevant models, which have improved our understanding of how the known clinical features of NEC could lead to the manifestations of disease. More recently, and in response to the complex nature of animal models, which often require interventions in tiny newborn animals, various laboratories – including our own – have turned to the development of *ex vivo* experimental systems to study NEC, so called ‘NEC-in-a-dish’ approaches (Werts et al., 2019). This Review will describe the current experimental models available for the study of NEC and their role in elucidating the important role of toll-like receptor 4 (TLR4) in disease pathogenesis, and will consider the key opportunities for research in the NEC field and how these may be best answered through precision-based models including the novel NEC-in-a-dish approach.

## Patient risk factors for NEC: from clinical observation to model development

The successful development of animal models for NEC research has been achieved in part through the identification of known patient risk factors for disease development, and then incorporating these risk factors into various models of disease pathogenesis. To this end, the most important factors associated with the development of NEC include prematurity and the administration of formula-based feeds (Chen et al., 2019; Hackam et al., 2019; Hällström et al., 2007), while the most important and effective protective factor is the administration of breast milk (Bode, 2018; Good et al., 2009). Large-scale epidemiological studies have identified additional factors that increase the risk of NEC, including blood transfusions (Mohamed and Shah, 2012) and the use of antacids (Christensen et al., 2010; Guillet et al., 2006; More et al., 2013). In contrast, a reduction in NEC severity has been linked to the administration of certain strains of probiotics (Alfaleh and Anabrees, 2014; Lau and Chamberlain, 2015; Patel and Underwood, 2018), feeding with donor breast milk (Buckle and Taylor, 2017; Cacho et al., 2017; Kantorowska et al., 2016; Quigley et al., 2018), adherence to strict feeding protocols (Oddie et al., 2017; Viswanathan et al., 2015) and limiting the use of prolonged empiric antibiotics (Gephart et al., 2017; Cotten et al., 2009; Patel et al., 2017). These risk factors have been incorporated into various animal models to provide a platform for understanding disease development. Because there are no available models of ‘spontaneous NEC’ or genetic variants that are known to give rise to NEC in mice or other animals, NEC must be induced experimentally. The most frequently used animal models of NEC are rats, mice and piglets, as we describe below, although rabbits and quails have been used in isolated studies in the past, probably due to the less-frequent availability and experience of researchers working with those animals (Ares et al., 2018; Bozeman et al., 2012; Good et al., 2014; Pisano et al., 2019; Waligora-Dupriet et al., 2009).

### Precision-based modeling of NEC: definitions and applications

As discussed above, NEC is a complex disease with significant variability in how patients present and what their outcomes are. In order to understand its pathogenesis, many investigators have turned to animal models designed to mimic the clinical manifestations of NEC, while also providing opportunity to test various potential therapies (Lu et al., 2014a). Although the currently used models have led to a number of important discoveries in the pathogenesis of NEC as described below, the fact that the overall survival of NEC patients has remained unchanged since the disease was first described in the 1970s (Bell et al., 1978) illustrates that there exists significant opportunity to refine our experimental models. Such refinements may allow for the study of NEC with greater precision, leading us to offer the term ‘precision-based modeling of NEC’.

From a definitional perspective, precision-based modeling of NEC represents a future generation of NEC models in which we incorporate techniques that more precisely mimic human disease and thus allow investigators to bridge the gap between translational discoveries and clinical application. We have defined precision-based models of NEC as models that incorporate the following four principles: (1) application of known clinical risk factors, including diet and prematurity; (2) validity – that the model closely mimics the phenotype of human disease; (3) inclusion of known and relevant influencers on disease development, including genomics, microbiomics and metabolomics; and (4) the ability to incorporate and test human-derived tissue from infants with and without NEC.

Of all the factors involved in precision-based modeling of NEC, it is our position that the incorporation of human-derived tissue is likely to be the most important determinant of the ability of these models to have an impact on our understanding and our treatment of disease. Although the lack of human tissue does not invalidate purely animal-based research, we posit that the very presence of human tissue in NEC models adds a level of precision and applicability to NEC that is simply not possible with animal models alone. In the subsequent paragraphs, in which animal models are evaluated, we will specifically discuss their ability for precision modeling of NEC according to the four criteria above.

### Experimental NEC in mice and the role of TLR4

Currently, the most commonly used animal models of NEC are formula-gavage newborn mouse models. The formula-gavage mouse model used in the authors' laboratory incorporates the important clinical features of NEC described above (prematurity, formula feeding, intermittent hypoxia) and has the chief advantage of offering the opportunity to interrogate the effects of various genetic and environmental alterations (Sodhi et al., 2008). Table 1 provides a summary of the most commonly used animal models of NEC and the degree to which they adhere to the four precision modeling criteria outlined above.

**Table 1.**
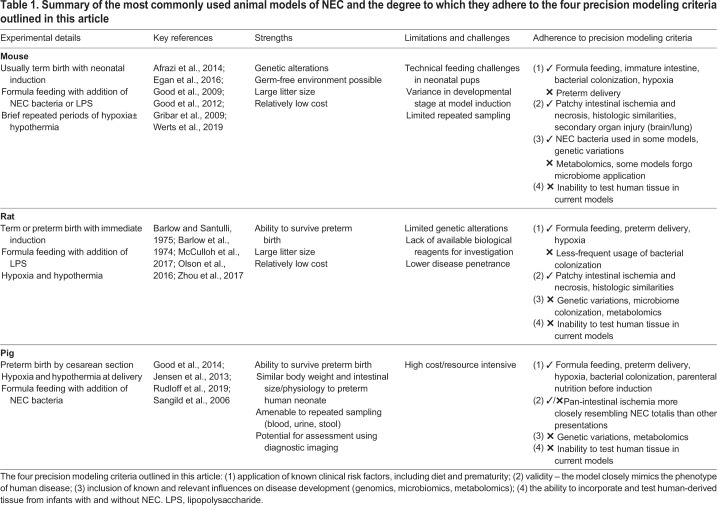
**Summary of the most commonly used animal models of NEC and the degree to which they adhere to the four precision modeling criteria outlined in this article**

**Fig. 2.**
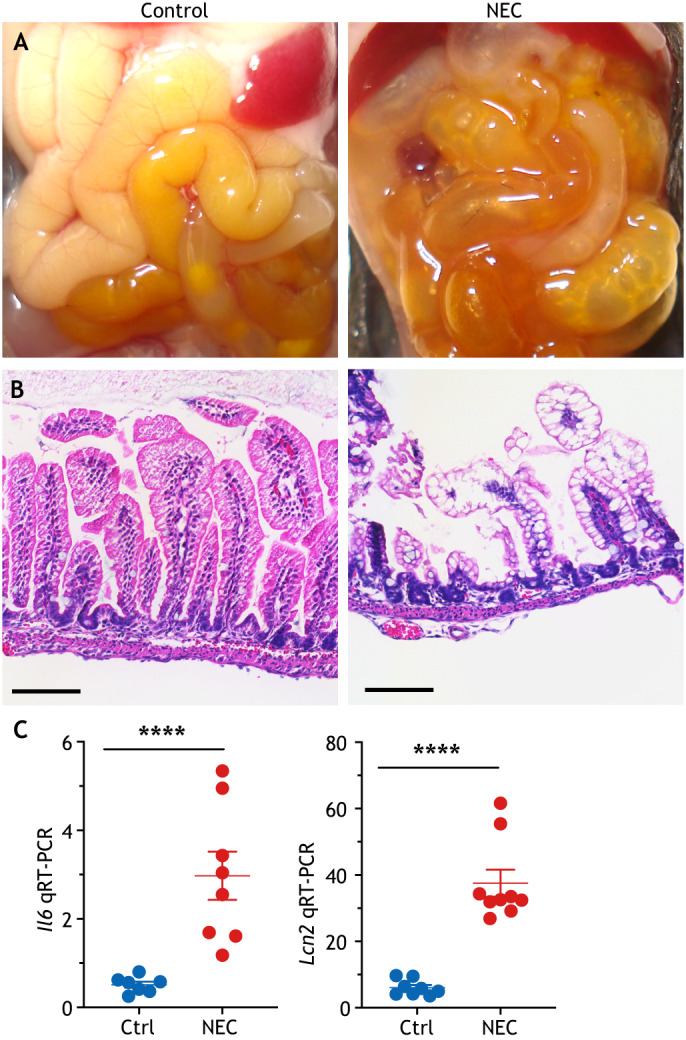
**Experimental NEC in mice.** (A) Gross morphology of the ileum showing edema, inflammation and pneumatosis. (B) Histologic appearance of the terminal ileum, showing submucosal separation, lamina propria edema and loss of villi. (C) Representative quantitative reverse transcription polymerase chain reaction (qRT-PCR) showing the expression of the inflammatory cytokine interleukin 6 (*Il6*) and lipocalin 2 (*Lcn2*). ****P<0.001; each dot represents an ileum sample from a separate mouse; error bars indicate s.e.m. Ctrl, control. Scale bars: 100 μm. Image credit: C.P.S.

The 4-day newborn mouse model begins with term delivery and NEC induction on postnatal day (P)7, based upon our observation that earlier induction carries significant mortality due to the technical challenges associated with handling tiny pups. Mouse pups are separated from their mother and housed in a neonatal incubator to prevent breastmilk feeding. The pups are instead gavage-fed formula five times daily (2:1 Abbott Nutrition Similac Advance infant formula and PetAg Esbilac caninine milk replacer, 43 ml/kg/feed), colonized with enteric bacteria isolated from the stool of an infant with NEC and subjected to brief periods of hypoxia twice daily (10 min at 95% N_2_, 5% O_2_). This model, which was validated in numerous studies, induces a pattern of features resembling human NEC, including patchy ileal necrosis, inflammation, luminal dilation and edema, accompanied by histological features of epithelial sloughing, necrosis and edema (Egan et al., 2016; Good et al., 2012; Werts et al., 2019) (Fig. 2).

**Fig. 3.**
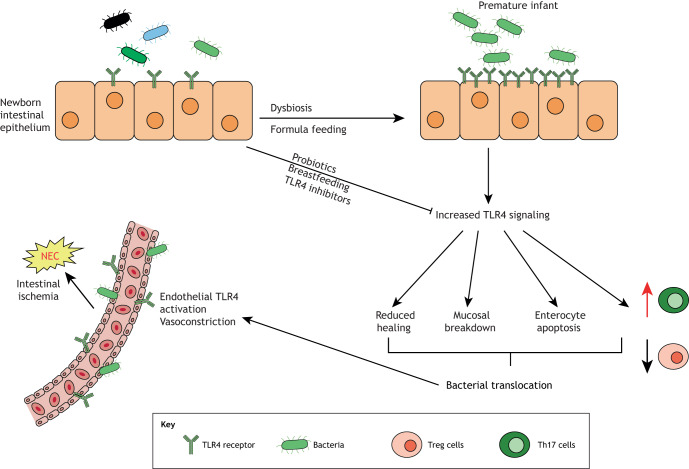
**The role of TLR4 activation in the pathogenesis of NEC.** Premature infants have elevated intestinal TLR4 expression compared to full-term infants. TLR4 becomes activated by colonizing Gram-negative microbes, resulting in significant mucosal inflammation, impaired epithelial repair and mucosal barrier breakdown, which permits bacterial translocation, further activating TLR4 on the intestinal mesenteric endothelium, and resulting in intestinal hypoperfusion and ischemia, leading to NEC. TLR4 activation can be inhibited by breast milk, probiotics or pharmacological inhibitors. Th17 cells, T-helper 17 cells; TLR4, toll-like receptor 4; Treg cells, regulatory T cells.

The key success of the hypoxia/formula gavage/bacteria NEC mouse model has been to link genetic risk factors to NEC induction, and therefore to increase the opportunity for precision modeling of NEC as described in Table 1. The clinical features of this model have allowed for the development of targeted NEC treatments and the discovery of how the innate immune system is involved in the pathogenesis of the disease. As first demonstrated in the mouse model, the innate immune receptor TLR4, which is activated by the structural component of Gram-negative bacteria lipopolysaccharide (LPS), is a critical component of NEC pathogenesis (summarized in Fig. 3). Specifically, our group (Leaphart et al., 2007) and others (Jilling et al., 2006) have shown a crucial role for TLR4 activation in the intestinal epithelium for the pathogenesis of NEC, which was further confirmed in mice lacking *Tlr4* specifically in the intestinal epithelium (Sodhi et al., 2012). Further work has shown that TLR4 expression is higher in the premature as opposed to the full-term intestine as a consequence of the role of TLR4 in regulating normal gut development through its upstream role in Notch signaling (Afrazi et al., 2014; Sodhi et al., 2012). Consequently, in the premature infant, in whom intestinal TLR4 expression is persistently high due to its ongoing developmental state, TLR4 becomes activated by the gut-colonizing Gram-negative microbes, resulting in significant mucosal inflammation (Hackam et al., 2019). The breakdown of the mucosal barrier then permits bacterial translocation (Hackam et al., 2013; Leaphart et al., 2007; Neal et al., 2013a), which activates TLR4 on the intestinal mesenteric endothelium (Yazji et al., 2013), resulting in loss of endothelial nitric oxide synthase, intestinal hypoperfusion and intestinal ischemia leading to NEC. Additionally, the TLR4-mediated inflammatory environment induces the influx of pro-inflammatory lymphocytes into the intestinal mucosa, which leads to further barrier injury (Egan et al., 2016).

Thus, the hypoxia/formula gavage/bacteria mouse model has demonstrated that NEC is a disease of exaggerated intestinal mucosal TLR4 signaling, a mindset that has paved the way for the development of targeted approaches for prevention and treatment. To this end, our group identified a novel family of NEC therapeutics that target TLR4 and that have been shown to prevent NEC in the hypoxia/formula gavage/bacteria NEC mouse model (Neal et al., 2013b; Wipf et al., 2015).

### Advances from mouse models

It is important to point out that different laboratories incorporate variations to their mouse models of NEC. For instance, the initial mouse model was developed in 2006 by the Caplan laboratory, and involved preterm delivery, gavage formula feeds every 2 h, brief hypoxia by 100% N_2_ and the addition of periods of hypothermia (Jilling et al., 2006). Refinements to the model in our laboratory have included term delivery, modifications in the type and frequency of feeds, and the use of adjuncts including live bacteria from a human infant with NEC (Werts et al., 2019), thus increasing the precision modeling capability. Other laboratories have adapted the mouse model further to include the administration of LPS (Miyake et al., 2016) or hypothermia (4°C for 10 min every 12 h as in the Besner model) (Wei and Besner, 2015; Wei et al., 2015) as important stressors to induce disease. In addition to revealing a role for TLR4 in NEC pathogenesis, these mouse models have been used to shed light on the key roles of probiotics (Athalye-Jape et al., 2018), human milk oligosaccharides (HMOs) (Good et al., 2016) and heparin-binding epidermal growth factor (Radulescu et al., 2010) in NEC. Our laboratory has shown that the DNA from probiotic bacteria inhibits TLR4 signaling through upregulation of TLR9, explaining in part the bacteria's protective effects in various cohort studies (Barbian et al., 2019; Good et al., 2014; Gribar et al., 2009) and clinical trials (Dermyshi et al., 2017; Lin et al., 2008). Our group has also used the mouse model to describe the mechanisms involved in secondary lung (Jia et al., 2016) and brain (Niño et al., 2018) injury that occurs in NEC patients, as well as to test novel diagnostic tools for the early detection of disease that are now being studied in humans (Goldstein et al., 2018).

Most recently, mouse NEC models have shown the importance of immunoglobulin A (IgA) for protecting against NEC in preterm infants (Gopalakrishna et al., 2019), validated the consequences of Paneth cell depletion in NEC (Lueschow et al., 2018), and begun to explore the mechanism underlying anemia and red blood cell transfusions in NEC (MohanKumar et al., 2019). These recent advances have incorporated a variety of modifications in the Caplan laboratory mouse model of NEC, to further elucidate the pathogenesis of the disease. For instance, Gopalakrishna et al. (2019) utilized a mouse NEC model very similar to that described above involving induction at 7-8 days of life, enteric colonization with *Enterobacter* and *Enterococcus*, and brief periods of hypoxia twice daily (5% O_2_, 95% N_2_). However, they replaced the typical formula feeding with breastfeeding by genetically altered dams that either could or could not produce IgA. They found that NEC could only be induced in the pups fed by dams that were deficient in IgA production, indicating the importance of maternal breast milk-derived IgA in preventing experimental NEC. The McElroy group used both chemical ablation and inducible genetic knockout to determine the role of Paneth cell depletion in the pathogenesis of NEC. They found that eliminating Paneth cells in P14-P16 mice resulted in NEC-like intestinal injury and a shift in the microbiome towards *Enterobacteriacea* species (Lueschow et al., 2018), similar to human NEC. The Maheshwari group designed a neonatal murine model to investigate the role in NEC of anemia and red blood cell transfusion, which are clinically associated with human NEC, although the mechanisms underlying this relationship are not well understood (MohanKumar et al., 2019). In a remarkable technical advance, they phlebotomized and transfused neonatal pups on P2, P4, P6, P8 and P10, and assessed for intestinal injury after colonization with *Serratia marcescens*. This study revealed that red blood cell transfusions triggered NEC-like intestinal injury in anemic pups through a TLR4-dependent immune response (MohanKumar et al., 2019).

While mouse models of NEC are clearly useful, they do have disadvantages that limit their broad applicability. Specifically, although survival at term is high, earlier delivery (Premkumar et al., 2014) is associated with high mortality (McCarthy et al., 2018). Moreover, because NEC induction requires eliminating breastfeeding and relying on infant formula for nutrition, this inevitably involves maternal separation, which can induce neonatal stress and impair normal development (Fabricius et al., 2008; Thomas et al., 2016). Furthermore, administration of enteral formula to a newborn mouse pup that is normally suckling requires gavage feeding, which is technically challenging in newborn animals that weigh 3 g or less (Ares et al., 2018). There are also biological limitations to various mouse models. For instance, those that rely on ablation of Paneth cells (Lueschow et al., 2018), intestinal ischemia/reperfusion (Clark et al., 1988; Musemeche et al., 1995), administration of epithelial toxins (Gonzalez Crussi and Hsueh, 1983; Namachivayam et al., 2017; Sun et al., 1995) or destruction of enteric glia (Bush et al., 1998) may induce an intestinal morphology that shares some similarities with NEC, but lacks essential clinical features (i.e. formula feeds, intermittent hypoxia that reflects immature lung development and microbial colonization), and are thus somewhat less translationally relevant to typical NEC, which occurs in formula-fed preterm infants (Cai et al., 2011). However, these models may have more value in uncovering the underlying mechanisms that lead to NEC in the subset of term and breastfed patients who develop the disease, or in those cases in which TLR4 activation may not be the inciting event, such as in cases of viral, fungal and Gram-positive NEC that have been reported (Coggins et al., 2015). Therefore, although neonatal mouse models of NEC are available and readily used (Ares et al., 2018; Lu et al., 2014a; Sodhi et al., 2008), they are associated with a significant learning curve, are technically challenging and suffer from a high associated technical mortality. These factors have led to the development of models in larger animals, including rats and pigs.

### Experimental NEC in rats

The original animal model of NEC as described by Barbara Barlow was developed in rats (Barlow and Santulli, 1975; Barlow et al., 1974), in which neonatal pups were fed formula and subjected to periods of hypoxia. A nutritive formula was developed to mimic rat breast milk, which was fed by dropper four times daily, and hypoxia was administered by placing animals in a sealed plastic bag once daily until they became limp and cyanotic (Barlow et al., 1974). This model led to the demonstration that breast milk protects against NEC (Barlow et al., 1974; Caplan et al., 1994), and also identified a role for nitric oxide in the pathogenesis of NEC (Nadler et al., 2000), the importance of bacterial colonization and TLR4 activation (Jilling et al., 2006), and the protective effects of beneficial commensal bacteria (Caplan et al., 1999), epidermal growth factor (Dvorak et al., 2002) and HMOs (Jantscher-Krenn et al., 2012). More-recent studies using the rat NEC model have identified mechanisms underlying intestinal barrier dysfunction (Ares et al., 2019), the protective effects of the hormone ghrelin (Meister et al., 2019), thrombomodulin (Li et al., 2019a) and fecal microbiota transplantation (Prado et al., 2019), and the deficiency of intestinal alkaline phosphatase that occurs in experimental NEC (Rentea et al., 2019), a finding that is also seen in human disease (Heath et al., 2019).

Additionally, the rat model has been used to develop several translational therapeutic strategies. The Besner group used the established rat NEC model that involves premature delivery by cesarean section, oral gavage formula feeding every 4 h, oral administration of LPS, and brief periods of hypoxic and hypothermic stress (McCulloh et al., 2017) to test the beneficial properties of various stem cell preparations in protecting against NEC. Intraperitoneal injection of amniotic fluid-derived mesenchymal stem cells, amniotic fluid-derived neural stem cells, bone marrow-derived mesenchymal stem cells and neonatal enteric neural stem cells reduced the incidence of NEC, indicating a potential role for cell-based therapy. The same group has demonstrated the efficacy of intraperitoneal administered exosomes secreted from bone marrow-derived mesenchymal stem cells in protecting against NEC development (Rager et al., 2016). Moreover, the group has used the rat model to develop and test a biofilm-based delivery system to simplify and improve the effectiveness of probiotic delivery for NEC prevention (Olson et al., 2018).

The rat NEC model is attractive because rats are inexpensive, have large litter sizes and are readily available (Lu et al., 2014a). The major weaknesses include limited ability to induce genetic alterations and the fact that the rat appears markedly more sensitive to endotoxin compared to certain model strains of mice, raising concerns regarding its applicability to human disease (Chang and Ohara, 1994; Copeland et al., 2005).

### Experimental NEC in pigs

**Fig. 4.**
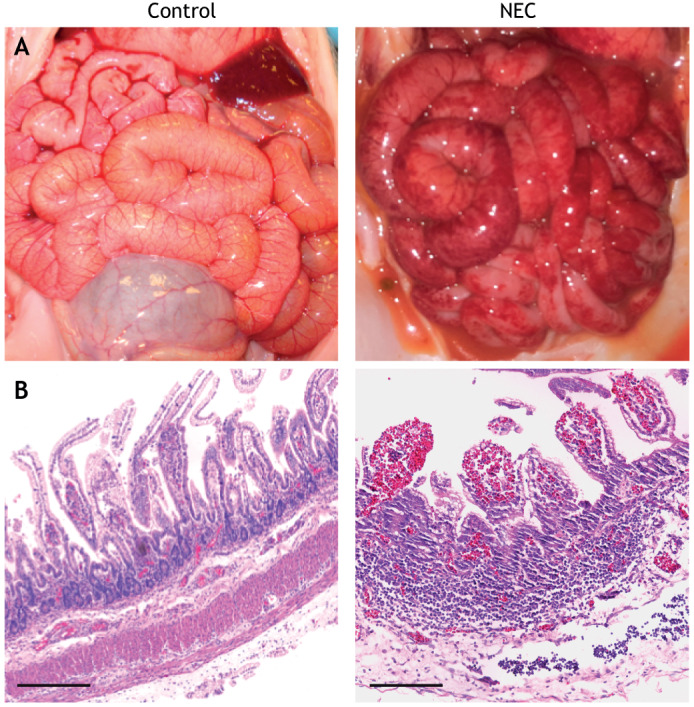
**Gross and histologic appearance of NEC in a piglet.** (A,B) The appearance of the small bowel (A) and histologic appearance of the terminal ileum (B) in the 4-day piglet model of NEC. Scale bars: 1 mm. Image credit: C.P.S.

Although rodents are the most frequently used animal models in NEC research, the piglet model may more closely resemble human disease. Piglets can be delivered preterm at 90-92% full gestation (Ares et al., 2018; Sodhi et al., 2008), with almost all of the premature litter surviving with basic supportive care (Fig. 4). These premature piglets can then be gavage fed formula, eliminating any exposure to breast milk, and the early preterm delivery by cesarean section adds both hypothermic and hypoxemic stress that mimics the clinical scenario of NEC. The piglet model reliably develops intestinal inflammation and NEC-like injury in the small and large intestine (Fig. 4) (Sangild et al., 2006). Variations on this model include parenteral nutrition for up to 48 h before initiation of enteral feeds (Call et al., 2018; Robinson et al., 2018b; Zamora et al., 2015), which adds significant clinical relevance as parenteral nutrition is a standard intervention in the first days of life for human infants born very prematurely and at high risk for NEC (Jensen et al., 2013; Zamora et al., 2015). The piglet model offers the advantage of using a large animal that has a large litter size with viable premature offspring, and the size of the piglet and histology of the intestine closely match those of premature infants (Sangild et al., 2013) and have comparable physiology (Lu et al., 2014a). The translational power of the piglet model has resulted in studies that have demonstrated the protective effects of probiotics (Good et al., 2014), colostrum (Sangild et al., 2006), breast milk (Jensen et al., 2013) and glucagon-like peptide 2, a gastrointestinal peptide with 1/3 sequence homology to glucagon (Benight et al., 2013), and have confirmed the importance of T-cell mucosal immunity (Anttila et al., 2003), reactive oxygen species (Koivusalo et al., 2002) and microvasculature disruption (Van Haver et al., 2008) in NEC. Additionally, work in the piglet model has demonstrated the capability of near-infrared spectroscopy to detect intestinal ischemia, which has been explored as a noninvasive modality to detect NEC onset earlier than the current imaging and clinical modalities can, with variable success (Chen et al., 2018). These studies suggest the potential for clinical translation of near-infrared spectroscopy; for instance, in infants who are suspected of having NEC, but have inconclusive imaging studies and/or an atypical clinical course. Prospective randomized trials comparing standard diagnostic criteria for NEC with near-infrared spectroscopy will be required to determine the usefulness of this promising technique.

Despite these translational advantages, the use of piglets for NEC research has significant limitations. The model can be prohibitively expensive and requires a holding area to care for a large pregnant sow, specific lifting equipment to transport the sow and extensive resources to humanely care for a litter of premature, messy, noisy, abundantly stooling piglets with NEC. There are also specific questions raised regarding the differences in findings from pig NEC models versus the human disease or studies in mouse models, including the roles of TLR4 (Østergaard et al., 2015), various HMOs (Rudloff et al., 2019) and probiotics (Cilieborg et al., 2011). Taken together, the piglet model is extremely suitable for studies that require a large animal mimic of the human disease, such as for drug or device testing, and for uncovering the pathophysiological mechanisms of the disease.

## Novel NEC-in-a-dish model platforms

Cell culture systems that allow for the three-dimensional growth of intestinal tissue and that also contain both epithelial and mesenchymal elements are termed organoids (Tauriello et al., 2018; Vlachogiannis et al., 2018), and have been developed in the past decade for – among other applications – the study of NEC (Ootani et al., 2009; Sato et al., 2009). Organoids can be derived from human intestine harvested at the time of resection for NEC or from age-matched control infants undergoing surgery for another indication (Dedhia et al., 2016; Spence, 2018; Stelzner et al., 2012). Additionally, organoids can be derived from primary intestinal tissue of animals subjected to experimental NEC in order to control for genetic background and environmental factors that are difficult to replicate between human patients. These primary organoid systems offer important advantages over cultured cell lines (Cetin et al., 2004), which may need to be transformed in order for them to grow in culture, typically grow in a two-dimensional monolayer and are susceptible to contamination by other cell lines (Routray et al., 2016). Organoids can be grown into a single cell type (termed enteroids when representing the intestinal epithelium, for example) or multiple cell types of an organ (termed intestinal organoids when referring to the intestine) (Stelzner et al., 2012).

Organoid and enteroid development, culture and treatment protocols follow a series of well-established steps that, when followed in a protocol, can optimize reproducibility. Enteroids can be derived by one of two stem cell-based methods: (1) induced pluripotent stem cells and (2) adult stem cells from intestinal crypts isolated from either surgical or biopsy specimens in the case of humans, or harvested tissue in the case of animals (Foulke-Abel et al., 2014). The stem cells are grown in conditioned cell culture media to encourage proliferation and differentiation through a variety of growth factors as established by Sato et al. (2011). After the enteroid culture is established, enteroids can be plated within an extracellular matrix-like scaffold, e.g. Matrigel (Westnet, #356235) and used for experimentation.

In our own group's recently described NEC-in-a-dish model, we subjected primary enteroid cultures to NEC-like conditions, including periods of hypoxia and treatment with bacteria cultured from a patient with severe NEC (Werts et al., 2019). Specifically, experiments were conducted on differentiated enteroids cultured in antibiotic-free media containing advanced Dulbecco's modified Eagle medium F12 supplemented with GlutaMax, B-27 supplement minus vitamin A, gastrin, N-acetyl-L-cysteine, noggin, A83-01 and epidermal growth factor for 36-72 h. Hypoxia was delivered in a cell culture incubator (5% CO_2_, 1% O_2_, 37°C) for 12 h, and the NEC bacteria added to the culture were prepared by diluting bacterial isolate from the stool of an infant with NEC and grown to log phase in LB broth. Dose adjustments for both intensity and duration of NEC-like condition exposures were performed to optimize the NEC-in-a-dish model to mimic human disease. It is noteworthy that these enteroids can be maintained in culture for several weeks, and that after several days they form complex cystic structures with a well-defined epithelial lining that is polarized and highly amenable to imaging and staining. Given their robust size (100 µm to mm), and the ease with which apoptosis, proliferation and cytokine release can be measured (Neal et al., 2012), enteroid cultures readily lend themselves to drug screens.

**Fig. 5.**
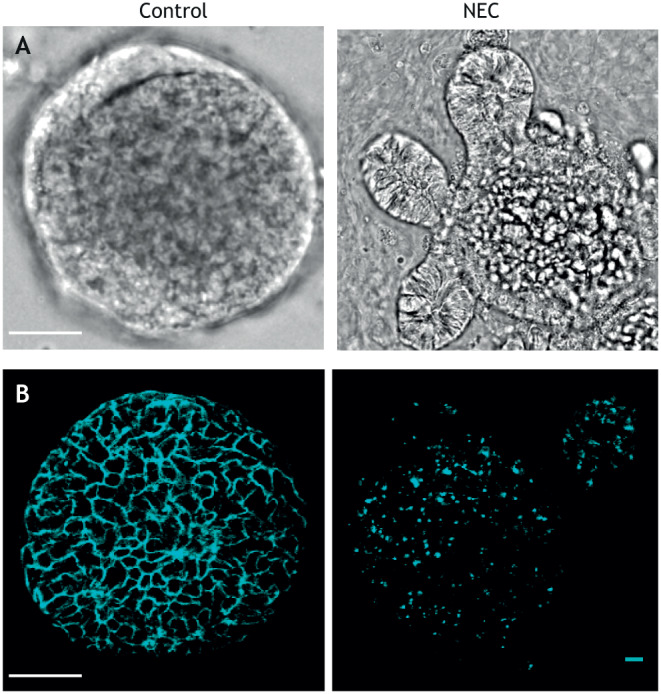
**NEC-in-a-dish model.** (A) Micrograph showing enteroids grown under NEC-inducing and control conditions. (B) Confocal micrograph showing enteroids with E-cadherin (cyan) to reveal epithelial architecture. Scale bars: 20 µm. Image credit: C.P.S.

The enteroids of this NEC-in-a-dish model reveal a pro-inflammatory cytokine response, architectural disruption and cell death similar to that seen in human disease (Werts et al., 2019). Strikingly, these effects can be mitigated by the addition of human breast milk and HMOs to the growth medium (Werts et al., 2019), which have been previously shown to protect against NEC (Autran et al., 2016; Bode, 2018; Good et al., 2016; Lu et al., 2014b) (Fig. 5). Interestingly, exposing enteroids to hypoxia or NEC bacteria alone shows no such damage, an observation also seen in the murine models (Werts et al., 2019), indicating that both components are necessary for a precision-based *ex vivo* NEC model. Our group and others have used similar NEC-in-a-dish models to confirm the importance of TLR4 activation (Egan et al., 2016; Neal et al., 2012; Sodhi et al., 2012; Werts et al., 2019), to show the loss of tight junctions in the intestinal epithelium (Li et al., 2019b) and to understand the mechanisms underlying HMO protection (Wang et al., 2019; Werts et al., 2019; Wu et al., 2019) against NEC.

While the above description of enteroids specifies postnatal tissue, investigators have utilized similar approaches but incorporated fetal intestinal tissue, and such approaches may therefore more accurately mimic the premature state of the intestine of NEC patients (Lanik et al., 2018; Senger et al., 2018). For instance, Nanthakumar et al. (2000) examined fetal tissue explants to reveal that the fetal intestine has a pro-inflammatory predisposition. The pro-inflammatory nature of the fetal intestine was attributed to the high levels of TLR4 expression that are present during *in utero* intestinal development compared to term birth, at which point TLR4 levels in the intestine are low (Nanthakumar et al., 2000), findings that are consistent with our work (Niño et al., 2016). Given the importance of TLR4 activation in the pathogenesis of NEC, this finding helps to explain why NEC is most commonly a disease of premature infants, suggesting that there may be physiological importance to the developmental stage from which NEC-in-a-dish models are derived (Leaphart et al., 2007; Lu et al., 2014b). Our group has used fetal tissue in culture to map the ontogeny of TLR4 (Leaphart et al., 2007), endoplasmic reticulum stress molecules (Afrazi et al., 2014) and TLR9 (Gribar et al., 2009), which all vary by developmental stage and contribute to NEC pathogenesis.

The advantages of the NEC-in-a-dish approach include the ability to test human tissue, the opportunity to perform gene knockdown experiments, and the relative low cost and technical ease with which such experiments can be performed. Disadvantages include the lack of an immune component, which we have determined to be necessary for the development of NEC (Egan et al., 2016), as well as other circulating factors that remain absent but potentially critical. This limitation could be addressed in future iterations of the model by developing a tissue-engineered small intestine including the immune (Perez et al., 2002), enteric nervous (Workman et al., 2017), vascular (Grebenyuk and Ranga, 2019; Seiler et al., 2020) and other mesenchymal components (Spence et al., 2011), which have been achieved in other enteroid systems (Kovler and Hackam, 2019; Nakamura and Sato, 2018). Importantly, while the immature nature of organoids may be considered a disadvantage in modeling adult-onset diseases, this feature of organoids is notably advantageous in the study of NEC, a disease that typically affects premature infants.

### Evaluating the therapeutic benefit of precision-based NEC-in-a-dish models

As described above, NEC-in-a-dish models offer significant theoretical advantages over animal models, in large part because they can incorporate human tissue into experiments. Given that enteroids can maintain the properties of the source tissue from which they were derived while being maintained in culture, enteroid-based NEC models are well suited as a precision-based tool for the study of this disease. For instance, enteroids can be grown from biopsies or surgical specimens from patients with and without NEC at various gestational ages. Those that are obtained from patients in the developmental window during which NEC most commonly occurs will have been exposed to known risk factors for disease development, including prematurity, formula feeding, *in utero* inflammation or anemia (Hällström et al., 2007; Nolan et al., 2019), and allow for the evaluation of inflammatory cytokine responses or architectural disruption similar to those seen in human disease (Werts et al., 2019). Moreover, the NEC-in-a-dish models enable the inclusion of known and relevant genomic and microbiome profiles specific to the individual human patient, and thus allow for the determination of precision drugs by screening known compound libraries. Such models also eliminate the potential confounding effects of the host microbiome in animal models, allowing for colonization with NEC bacteria only. Thus, these NEC-in-a-dish experiments and the translational relevance of these approaches provide the potential of a personalized approach for the treatment of NEC at the bedside. Operationally, a clinical stream would consist of a sick neonate with NEC being assessed by an investigative team, performing a rectal biopsy at the bedside and transferring the tissue to a clinical laboratory, where intestinal enteroids are derived, which would express the clinical and genetic factors specific for that individual patient. After several days in culture, the enteroids would then be used in a screen of a known library of clinical compounds (Neal et al., 2013b), or co-cultured with certain probiotics and evaluated for specific readouts that may directly relate to clinical improvement; for example, proliferation as an indicator of healing versus apoptosis as an indicator of injury. In this manner, NEC-in-a-dish approaches can facilitate personalized modeling, which can lead to effective therapy for individual patients with NEC.

## Opportunities for NEC research using novel disease models

NEC remains a highly morbid disease with few specific treatment options. To develop new treatments, we posit that future research will require an emphasis on personalized, precision models that will include human-derived tissue. Such studies could allow the field to refocus efforts towards understanding the human disease rather than understanding the experimental disease in animals. Such a shift in focus need not ignore the integration of *ex vivo* cell-based platforms with animal models that incorporate features of the disease, but rather should combine *ex vivo* and *in vivo* models to develop and test potentially effective therapies. The complex interplay between developmental, nutritive, genetic and microbial influences will require machine learning to integrate all the factors that lead to NEC development. Moreover, given that NEC is usually a disease of premature infants, it stands to reason that advances will be made wherever models incorporate features of the developing intestine, necessitating close collaboration between developmental biologists and the NEC research community. Additional advances in NEC research may benefit from incorporation of those factors at the precise window of time during which NEC develops, with consideration given to the gestational age at disease onset rather than at birth. It is noteworthy that the community is already developing biobanks of tissue specimens resected from both NEC patients and otherwise healthy infants of the same gestational age, which promise to accelerate breakthroughs in determining the age-related and genetic predisposition to NEC in premature infants (Chaaban et al., 2019).

## Conclusion

NEC remains a devastating disease of premature infants, and decades of clinical and scientific research have yet to produce significant improvements in outcomes for the affected babies. Although large-scale epidemiologic studies have identified risk factors for NEC, and experimental models have yielded significant clues on the biological foundations of the disease, targeted therapeutic strategies are lacking. The future of NEC research will require a precision approach, involving the integration of *ex vivo* models that incorporate human tissue, along with animal models that take advantage of the ability to manipulate nutrient load, the immune system and the microbiome. Of equal importance will be to include research teams with expertise in mathematical modeling and machine learning, as well as scientists of varied backgrounds including developmental biologists, cell biologists, geneticists, immunologists, gastroenterologists, neonatologists and pediatric surgeons, who together will provide the fresh thinking required to solve this complex disease.
